# Deregulation of extracellular matrix modeling with molecular prognostic markers revealed by transcriptome sequencing and validations in Oral Tongue squamous cell carcinoma

**DOI:** 10.1038/s41598-020-78624-4

**Published:** 2021-01-08

**Authors:** Soundara Viveka Thangaraj, Vidyarani Shyamsundar, Arvind Krishnamurthy, Vijayalakshmi Ramshankar

**Affiliations:** 1 Department of Preventive Oncology (Research), Cancer Institute (WIA), Adyar, Chennai, 600 020 India; 2 Center for Oral Cancer Prevention and Research (COCPAR), Sree Balaji Dental College, Chennai, 600 100 India; 3 Department of Surgical Oncology, Cancer Institute (WIA), Adyar, Chennai, 600 020 India

**Keywords:** Cancer, Biomarkers

## Abstract

Oral Tongue Squamous Cell Carcinoma (OTSCC), a distinct sub-group of head and neck cancers, is characteristically aggressive in nature with a higher incidence of recurrence and metastasis. Recent advances in therapeutics have not improved patient survival. The phenomenon of occult node metastasis, even among the purportedly good prognosis group of early-stage and node-negative tongue tumors, leads to a high incidence of locoregional failure in OTSCC which needs to be addressed. In the current study, transcriptome analysis of OTSCC patients identified the key genes and deregulated pathways. A panel of 26 marker genes was shortlisted and validated using real-time PCR in a prospective cohort of 100 patients. The gene expression was correlated with clinicopathological features including occult node metastasis, survival, and therapeutic outcome. The up-regulation of a panel of 6 genes namely, matrix metalloproteinase 9 (MMP9), Laminin subunit Gamma 2 (LAMC2), Desmoglein 2 (DSG2), Plasminogen Activator Urokinase (PLAU), Forkhead Box M1 (FOXM1), and Myosin 1B (MYO1B) was associated with failure of treatment in the early stage (T1, T2). Up-regulation of Tenacin C (TNC) and Podoplanin (PDPN) was significantly correlated with occult node positivity. Immunohistochemical analysis of LAMC2, MMP9, and E-Cadherin (ECAD) confirmed these markers to be indicators of poor prognosis. We propose this panel of valuable prognostic markers can be clinically useful to identify poor prognosis and occult node metastasis in OTSCC patients.

## Introduction

Oral Tongue Squamous Cell carcinoma (OTSCC) represents a major portion of oral cavity cancers, especially in India. It is now evident that OTSCC needs to be studied as a separate entity and not be included in the broad scheme of oral cancers, as often reported—an approach that increases the power of the study but diminishes the credibility of the inference made from such studies. As per the data from National Cancer Registry Program (NCRP; www.ncdirindia.org), tongue cancer ranks 4th among the 10 leading sites of cancer among males in Chennai. There is a sharp increase in the incidence of tongue cancers not only in India^1^ but also in young white women^2^ and in Nordic countries^3^. Striking aspect of OTSCC epidemiology is the increase in incidence of OTSCC among young patients^4,5^ and women below 44 years of age^6, 7^.

OTSCC is an aggressive type of tumor with poor prognosis^8^ and the single most important aspect determining the staging, management and prognosis of patients with tongue cancers is the status of the cervical lymph nodes and occult node metastasis. However, the inaccuracy of clinical examination and imaging to reliably detect occult cervical lymph node micrometastasis has resulted in elective neck dissections becoming a standard of care for a vast majority of the OTSCC management, based on the evidences from randomised controlled trials^9^. The other clinicopathological factors predicting poor prognosis include increased depth of invasion^10,11^, increasing pT, the presence of extra-capsular extension^12,13^ and endophytic appearance of tumor^14^. In spite of the multiple attempts made to classify molecular markers in OTSCC with promising candidates emerging^15–19^, they have not been translated to clinics and therapeutic decisions are still largely driven by the classical clinico-pathological factors like tumor stage, patient’s age and performance status^20^ which may be insufficient. Though numerous biomarkers have been presented as useful prognosticators for OTSCC, they have been unsatisfactory with no possible reliable conclusions. The alarmingly high rates of loco-regional recurrences seen in OTSCC warrants molecular markers that can predict patients at higher risk of morbidity and mortality and to design appropriate treatment approaches in patients.

Transcriptome sequencing (RNA-seq) as a platform of analysis not only has a better sensitivity and dynamic range than traditional microarrays, but also enables identification of novel transcripts and more exhaustive study of biological pathways^21^. In the current study, transcriptome sequencing (RNA seq) was done to identify the differentially expressed genes (DEG), in a set of OTSCC samples with validations by qPCR and Immunohistochemistry to derive salient markers for prognostication of OTSCC patients in a prospective study cohort.

## Results

### Transcriptome sequencing

Clinical characteristics of patients (n = 12) studied by transcriptome sequencing is shown in Supplementary Table S1. An average of 9.8 Gb of data was generated with more than 91% of bases passing Q30 Phred score. We identified a total of 3,705 (2,610 up-regulated, 1095 down-regulated) differentially expressed genes (DEG) (FDR < 0.05 and *P* value < 0.05) in tongue carcinoma samples. The quality of the results depends on the number of genes analyzed, which in turn is based on cut-offs and statistical significance. Hence for further functional analyses, we filtered only highly significant 2073 DEGs (1578 up-regulated, 495 down-regulated) with *P* value < 0.01 and Fold Change > 10. The heatmap representing the expression of top 100 DEG in OTSCC was derived from results obtained by transcriptome sequencing (Supplementary Figure S1).

The top 15 over expressed and under expressed genes are listed in Table 1 and all the highly significant 2073 DEGs are listed in Supplementary Table S2. The top up-regulated genes included many genes from the Matrix metallopeptidase family (MMP—1, 3, 9, 10, 12 and 19) and Melanoma Antigen family (MAGE A–3,4 and 6). The top down regulated genes included many genes from the Keratin family (KRT—4, 13, 36, 72 and 78).

**Table 1:** Top 15 upregulated and down regulated genes in OTSCC identified by transcriptome sequencing.

Up regulated genes				Down regulated genes			
Gene name	Gene symbol	Fold change (Log2)	*P* value	Gene name	Gene symbol	Fold change (Log2)	*P* value
Matrix Metalloproteinase 12	MMP12	8.61	1.44E−26	Dopachrome tautormerase	DCT	− 7.69	4.40E−08
Matrix Metalloproteinase 9	MMP9	8.29	7.64E−22	Keratin 36	KRT36	− 7.52	4.99E−16
MAGE family member A3	MAGEA3	8.85	3.08E−17	Calmodulin like 6	CALML6	− 7.51	5.35E−14
MAGE family member A6	MAGEA6	8.57	8.77E−16	WNT inhibitory factor 1	WIF1	− 6.61	6.14E−05
Matrix Metalloproteinase 1	MMP1	8.81	1.93E−15	Myocilin	MYOC	− 6.56	2.10E−07
CXC motif chemokine ligand 1	CXCL1	7.28	2.93E−15	MT-CO1 pseudogene 3	MTCO1P3	− 6.52	1.10E−05
SPOC Domain containing 1	SPOCD1	7.05	2.04E−13	Tyrosinase	TYR	− 6.32	2.87E−06
Cyclic Nucleotide gated channel subunit Beta 1	CNGB1	7.69	1.12E−11	Paired Box 1	PAX1	− 6.16	3.31E−07
Inhibin subunit beta A	INHBA	7.40	1.56E−11	Cytochrome P450 Family 2 subfamily F member 3	CYP4F35P	− 6.15	9.69E−06
Matrix Metalloproteinase 10	MMP10	7.85	2.80E−11		RP11-641A6.8	− 6.15	1.37E−06
Matrix Metalloproteinase 3	MMP3	7.82	4.17E−11	Leucine rich repeats and transmembrane Domains 1	LRTM1	− 6.13	1.74E−09
C–C Motif chemokine Ligand 11	CCL11	7.57	1.18E−10	Keratin 72	KRT72	− 6.03	6.38E−11
Marginal Zone B and B1 cell specific protein	MZB1	7.01	8.81E−09	Serine Peptidase inhibitor-9	SPINK9	− 5.97	1.65E−05
MAGE family member A4	MAGEA4	6.97	1.60E−08	Dehyrogenase/reductase 7C	DHRS7C	− 5.87	5.43E−06
F2R like thrombin or trypsin receptor 3	F2RL3	6.80	2.85E−13	Keratin 4	KRT4	− 5.81	0.00E + 00

### Gene set enrichment analysis on DEG obtained by transcriptome sequencing reveals deregulated extracellular matrix (ECM) in OTSCC

To understand the biological roles of the DEG from OTSCC, we performed a gene set enrichment analysis on the DEG derived by transcriptome sequencing. We found GO terms for biological process enriched for Inflammatory response, Extracellular Matrix organization, Cell adhesion, Collagen Catabolic Process, Immune response and Angiogenesis. The top most significant enriched GO term for Cellular components were related to extracellular matrix remodeling. The top most significant enriched GO term for Molecular Function were Metalloendopeptidase activity, Heparin binding, Extracellular matrix structural constituent, Collagen binding and Receptor activity. The chief deregulated KEGG pathways included Cytokine-cytokine receptor interaction, Focal adhesion, ECM-receptor interaction and PI3K-Akt signaling pathways. The GO enrichment and KEGG pathway for differentially expressed genes (DEG) is shown in Supplementary Figure S2.

### Protein–protein interaction (PPI) network shows ECM and focal adhesion interaction pathways deregulated in OTSCC

The PPI network generated in STRING using the 1698 DEGs included 1392 nodes and 2516 edges with a PPI enrichment *P* value < 1.0e−16. The top 10 nodes are presented in Supplementary Table S3. The module analysis tool extracted 8 most important modules with MSCORE > 6 as sub-networks from the overall PPI network (Supplementary Table S4). The top hub genes, Amyloid Beta Precursor Protein (APP), G protein subunit beta 5 (GNB5) and G protein subunit Gamma Transducin 2 (GNGT2) were part of Module 1. Functional enrichment analysis of the top 2 modules identified the GO terms and KEGG pathways listed in Supplementary Table S5. Most significant module 1 was enriched for GO terms chemokine activity and G-protein coupled receptor binding pathway and for the KEGG pathways, Chemokine receptor signaling pathway and cytokine–cytokine interaction pathways. Module 2 showed enrichment of GO biological process terms Collagen catabolic process and Extracellular matrix organization while the KEGG pathways enriched consisted of ECM receptor interaction and Focal adhesion. Integrated sub network of the top most significant modules of OTSCC-PPI network is shown in Supplementary Figure S3.

### Validation of differentially expressed proteins by qPCR

**Figure 1:**
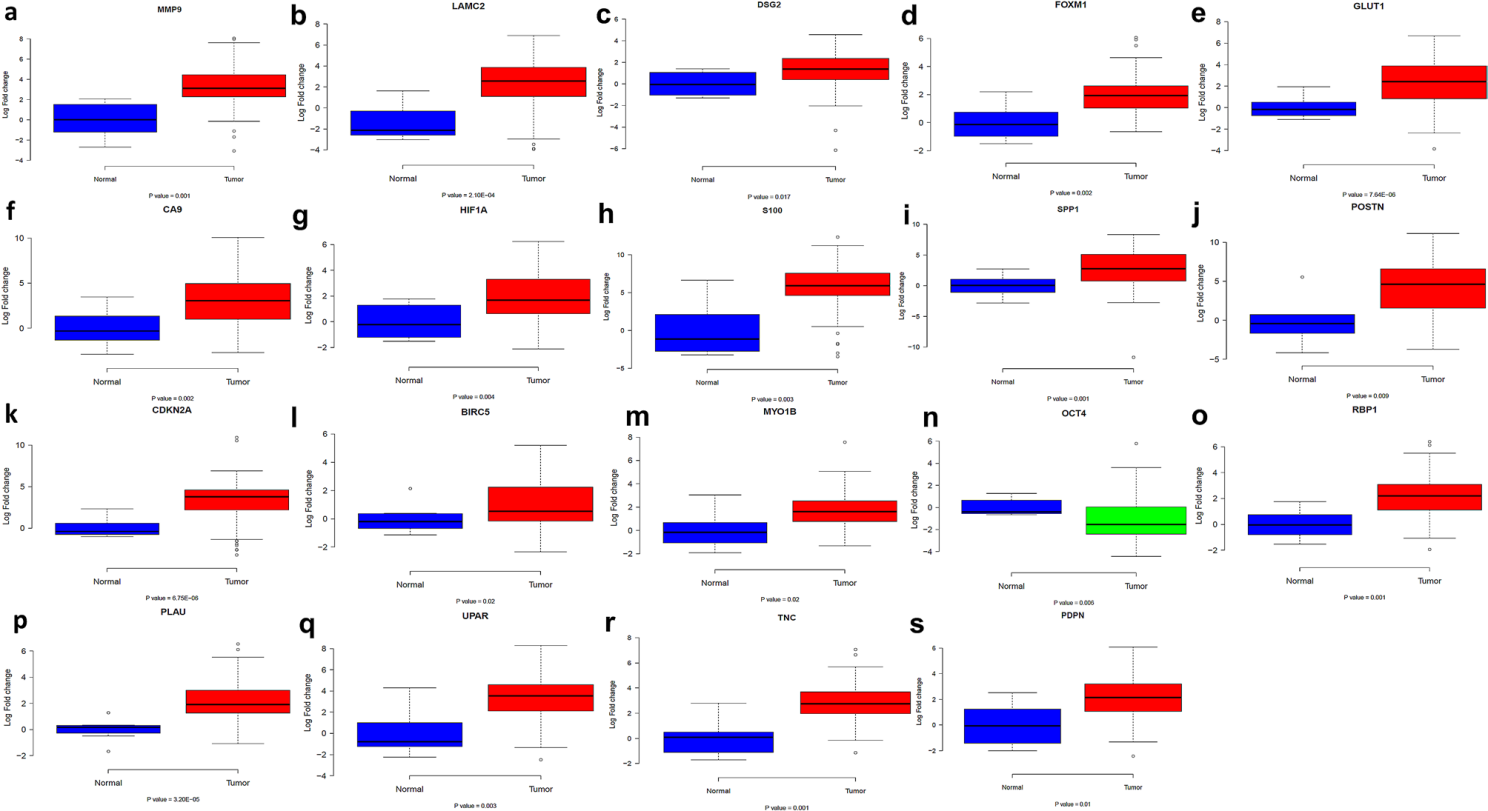
Box plot comparing the expression of genes in absolute normal tongue and OTSCC samples. Average log_2_ fold change from tumor and adjacent normal samples have been compared and the *P* value from an unpaired *t* test is indicated in the figure. (**a**) MMP9 (*P* = 0.001) (**b**) LAMC2 (*P* = 2.10e−04) (**c**) DSG2 (*P* = 0.017) (**d**) FOXM1 (*P* = 0.002) (**e**) GLUT1 (*P* = 7.64e−06) (**f**) CA9 (*P* = 0.002) (**g**) HIF1A (*P* = 0.004) (**h**) S100 (*P* = 0.003) (**i**) SPP1 (*P* = 0.001) (**j**) POSTN (*P* = 0.009) (**k**) CDKN2A (*P* = 6.75e−06) (**l**) BIRC5 (*P* = 0.001) (**m**) MYO1B (*P* = 0.02) (**n**) OCT4 (*P* = 0.006) (**o**) RBP1 (*P* = 0.001) (***p***) PLAU (*P* = 3.20 e−05) (**q**) UPAR (*P* = 0.003) (**r**) TNC (*P* = 0.001) (**s**) PDPN (*P* = 0.01).

Biomarkers for a qPCR-based validation in prospective OTSCC patients (n = 100) were chosen based on interrelationships assessed by Pearson correlation with respect to the genes involved in ECM remodeling and EMT. The markers chosen were LAMC2, TNC, PDPN, SPP1, PLAU, PLAUR, MMP9, FOXM1, VIM, CTNNB1, GLUT1, HIF1A, SOX2, OCT 4, VEGF, MYO1B, RBP1, SVN, CA9, DSG2, SURVIVIN, S100, TWIST2, CCND1, ECAD, POSTN. Figure 1 shows the box plot comparing the expression of genes in absolute normal tongue and OTSCC samples. We obtained 19 markers out of the 26 chosen to be significantly differentially expressed between tumor and adjacent, apparently normal tissue. We performed a qRT-PCR analysis of the 19 markers in tongue tumor and histologically normal tongue samples. There was a significant alteration in the mRNA expression of these markers between tongue tumor and normal tongue samples as depicted (Fig. 1). The biomarkers studied were based on having a significant association among the EMT markers—MMP9, SPP1, LAMC2, DSG2, S100 and TNC, hypoxia markers—HIF1α, CA9 and GLUT1 and other markers including POSTN, CDKN2A, UPAR, PLAU, PDPN, RBP1, MYO1B, BIRC5, FOXM1 and OCT4 (Table 2).

**Table 2:** Differentially expressed genes associated with OTSCC assessed by qPCR.

Gene name	Fold change (Log 2)	*P* value
MMP9	3.34	0.001
LAMC2	2.29	2.10E−04
DSG2	1.27	0.017
FOXM1	1.27	0.002
S100	5.67	0.003
GLUT1	2.35	7.64E−06
HIF1 α	1.90	0.004
CA9	3.19	0.002
SPP1	2.84	0.001
POSTN	4.27	0.009
BIRC5	1.10	0.021
CDKN2A	3.35	6.75E−06
OCT4	− 1.03	0.006
RBP1	2.16	0.001
PLAU	2.21	3.20E−05
UPAR	3.22	0.003
TNC	2.86	0.001
PDPN	2.04	0.01
MYO1B	1.75	0.015

The interrelationships derived is shown as follows. LAMC2 expression was most strongly correlated to TNC (r =  + 0.5, *P* value = 3.16E−08), PLAU (r =  + 0.5, *P* value = 4.22E−07), PDPN (r =  + 0.5, *P* value = 2.44E−08), SPP1 (r =  + 0.49, *P* value = 1.24E−07) and PLAU (r =  + 0.47, *P* value = 4.22E−07) while SPP1 correlated strongly to UPAR (r =  + 0.5, *P* value = 3.43E−08) and PLAU(r =  + 0.47, *P* value = 3.23E−07). MMP9 expression was positively related to FOXM1 (r =  + 0.49, *P* value = 1.02E−07) and TNC (r =  + 0.46, *P* value = 6.67E−07). VIM expression was strongly associated with CTNNB1 (r =  + 0.6, *P* value = 8.33E−12) while DSG2 was similarly correlated to TNC (r =  + 0.6, *P* value = 1.76E−10) and S100 to GLUT1 (r =  + 0.5, *P* value = 6.41E−08). TNC was positively related to PDPN (r =  + 0.5, *P* value = 1.16E−08), UPAR (r =  + 0.6, *P* value = 2.31E−12), HIF1A (r =  + 0.6, *P* value = 1.05E−09), PLAU (r =  + 0.5, *P* value = 4.61E−08) and MYO1B(r =  + 0.5, *P* value = 5.78E−09); PDPN to PLAU (r =  + 0.48, *P* value = 2.19E−07), UPAR(r =  + 0.5, *P* value = 5.06E−08) and MYO1B(r =  + 0.48, *P* value = 1.51E−07). The hypoxia marker HIF1A was correlated to the expression of SOX2(r =  + 0.56, *P* value = 2.56E−10), PLAU (r =  + 0.58, *P* value = 4.81E−11), FOXM1 (r =  + 0.58, *P* value = 4.17E−11), MYO1B (r =  + 0.53, *P* value = 4.37E−09), SVN (r =  + 0.47, *P* value = 2.48E−07) and GLUT1(r =  + 0.48, *P* value = 1.96E−07). TWIST2 was positively linked to SOX2 (r =  + 0.5, *P* value = 3.83E−08), OCT4 (r =  + 0.48, *P* value = 1.81E−07) and MYO1B (r =  + 0.47, *P* value = 3.22E−07); SOX2 strongly linked to OCT4 (r =  + 0.56, *P* value = 3.2E−10) and OCT4 to VEGF (r =  + 0.54, *P* value = 1.35E−09). Similarly, CA9 correlated with RBP1 (r =  + 0.53, *P* value = 4.89E−09), PLAU (r =  + 0.48, *P* value = 2.18E−07) and GLUT1 (r =  + 0.57, *P* value = 1.44E−10), OCT4 to VEGF (r =  + 0.54, *P* value = 1.35E−09) and SVN(r =  + 0.63, *P* value = 2.98E−13); RBP1 to PLAU (r =  + 0.69, *P* value = 2.13E−12), FOXM1 (r =  + 0.49, *P* value = 8.74E−08), SVN (r =  + 0.51, *P* value = 2.79E−08) and GLUT1 (r =  + 0.51, *P* value = 3.03E−08); PLAU to FOXM1 (r =  + 0.65, *P* value = 4.71E−14), MYO1B(r =  + 0.57, *P* value = 1.58E−10), SVN (r =  + 0.62, *P* value = 1.15E−12) and GLUT1 (r =  + 0.47, *P* value = 3.12E−07) and FOXM1 correlated to SVN (r =  + 0.51, *P* value = 2.5E−08) and GLUT1 (r =  + 0.59, *P* value = 1.13E−11). Moderately strong to weak significant correlations was observed among the biomarker expression in the tongue tumors and listed in Table 3.

**Table 3:**
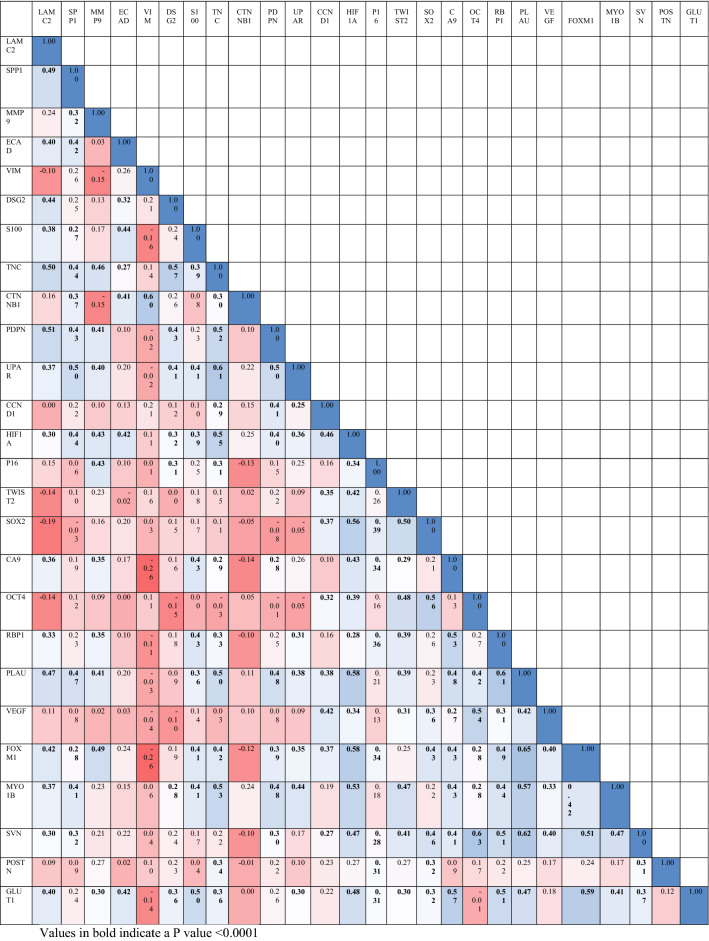
Inter-relationship between molecular markers in OTSCC as represented by Pearson’s correlation coefficient. Values in bold indicate a *P* value < 0.0001.

**Figure 2:**
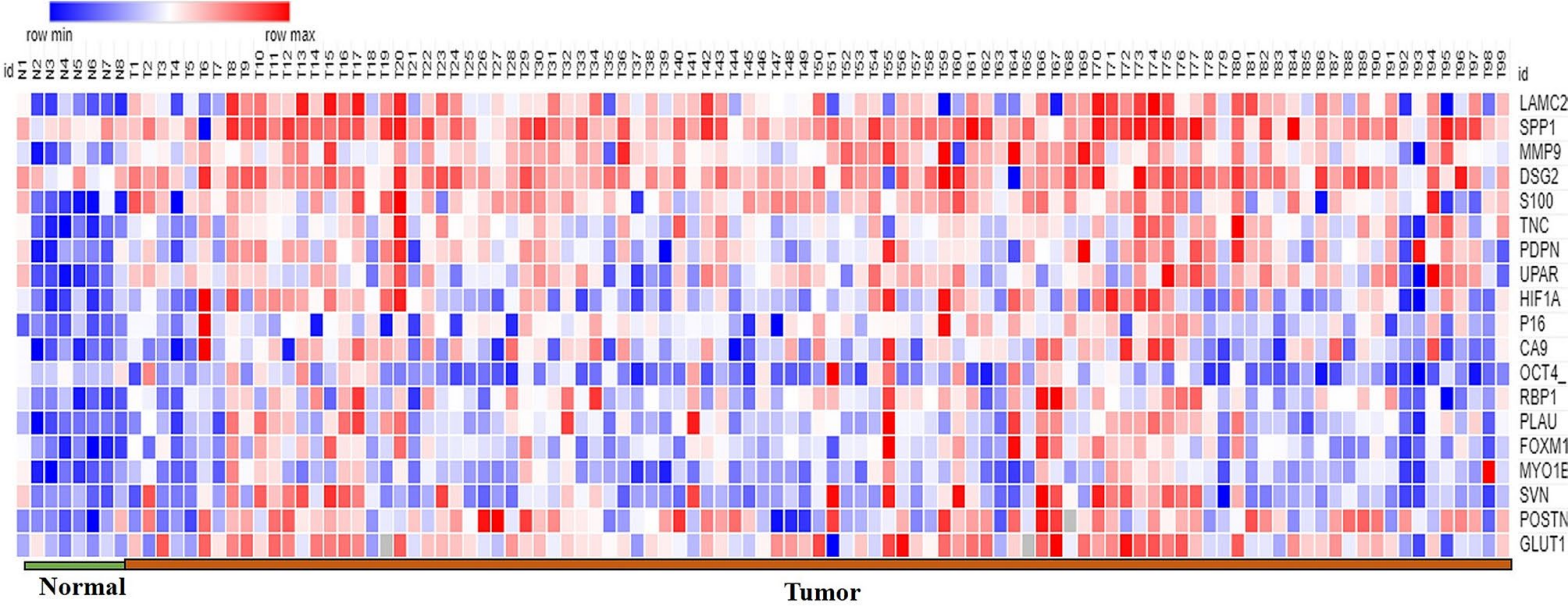
Heatmap representing the expression levels of the significant differentially expressed marker genes among the normal and OTSCC samples.

The clinical characteristics of prospective cohort of OTSCC patients used for validation studies (n = 100) is shown in Supplementary Table S6. Tissue extracts from OTSCC tumor and normal was prepared as described in Methods. The expression of the different markers was assessed for correlation with pathological stage, outcome and lymph node status. One patient was lost to follow-up and was excluded from the survival analyses. The same pattern of expression could be visualized in the heatmap of the biomarker expression as shown in Fig. 2 showing the expression levels of the significant differentially expressed marker genes among the normal and OTSCC samples.

### Panel of 6 molecular markers useful in predicting recurrence among patients in early staged OTSCC

In the early staged patients, treatment failed in 22.5% (9) cases and 77.5% (31) cases showed NED. Among the panel of 19 biomarkers studied, we identified a panel of 6 genes showing overexpression namely, MMP9 (*P* value = 0.02), LAMC2 (*P* value = 0.02), DSG2 (*P* value = 0.02), PLAU (*P* value = 0.02), FOXM1 (*P* value = 0.02) and MYO1B (*P* value = 0.02) to be associated with failure of treatment in the early stage patients (Table 4). No such panel could be derived for advanced stages of OTSCC.

**Table 4:** Molecular markers associated with stage, outcome and cervical node status.

	Gene name	*P* value	F value
Early stage versus advanced stage	LAMC2	0.016	14.32
	VIM	0.019	0.24
	HIF1A	0.012	2.39
	TWIST2	0.029	2.65
	SOX2	0.024	2.05
Cervical node positive versus negative	LAMC2	0.017	4.18
	VIM	0.001	0.001
	TWIST2	0.033	1.87
	SOX2	0.024	0.64
Occult node positive versus negative	TNC	0.049	6.76
	PDPN	0.049	0.5
Early staged OTCC prognostic panel	MMP9	0.016	0.1
	LAMC2	0.022	10.7
	DSG2	0.038	1.21
	PLAU	0.046	1.06
	FOXM1	0.008	0.99
	MYO1B	0.027	0.89

### Biomarkers depicting occult nodal and cervical nodal status

Overexpression of TNC (*P* value = 0.05) and PDPN (*P* value = 0.05) significantly indicated occult node positive status while the overexpression of LAMC2 (*P* value = 0.02), VIM (*P* value = 0.001), TWIST2 (*P* value = 0.03) and SOX2 (*P* value = 0.02) were the significantly associated with the cervical lymph node involvement.

### Molecular markers useful in differentiating the clinical stages in OTSCC

The relative expression of LAMC2 (*P* value = 0.02), VIM (*P* value = 0.02), HIF1A (*P* value = 0.01), TWIST2 (*P* value = 0.03) and SOX2 (*P* value = 0.02) were found to be significantly differentially expressed among the early and advanced stage tumors. The differential expression of potential markers between early versus advanced staged OTSCC has been shown (Supplementary Figure S4).

### Validation of protein expression by immunohistochemistry

**Figure 3:**
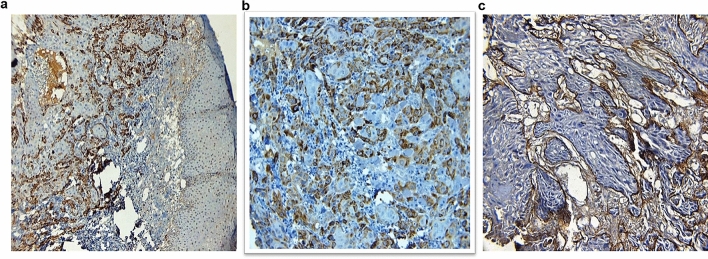
Immunoexpression of LAMC2: (**a**) WDSCC showing intense cytoplasmic positivity for LAMC2 10X. (**b**) WDSCC showing intense cytoplasmic positivity for LAMC2 20X. (**c**) MDSCC showing intense positivity for basement membrane, but cytoplasm in negative for LAMC2, 20X.

**Figure 4:**
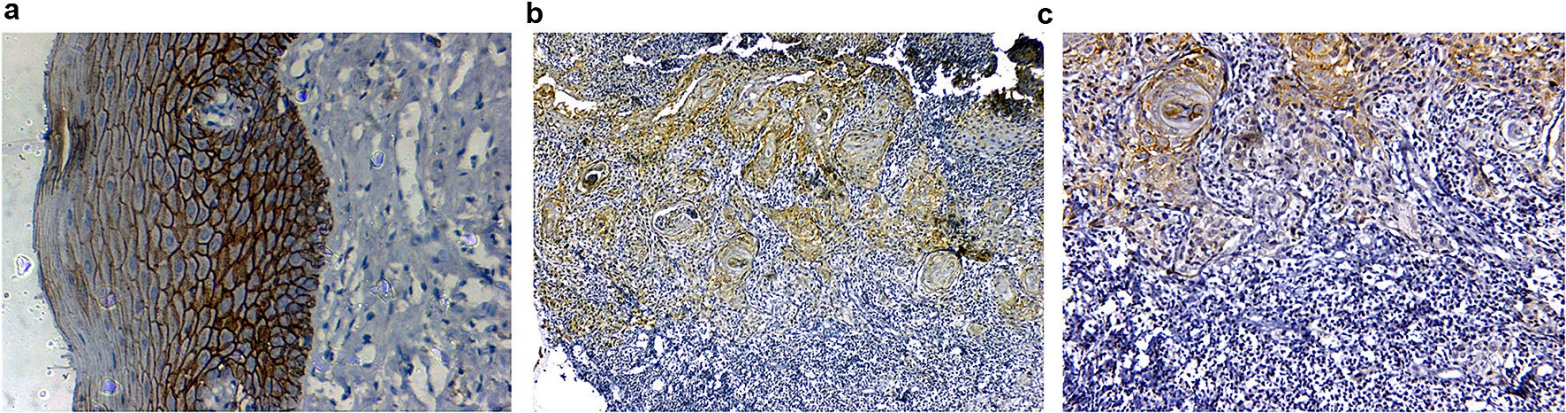
Immunoexpression of ECAD (**a**). Normal epithelium with intense E-cadherin positivity (**b**). Reduction of ECAD expression at ITF 10X (**c**). Reduction of ECAD expression at ITF 20X.

**Figure 5:**
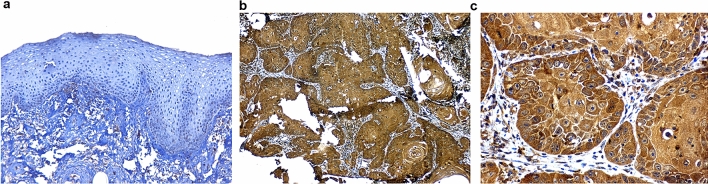
Immunoexpression of MMP9 (**a**). Normal mucosa showing minimum stain for MMP9 (**b**). WDSCC showing intense MMP9 stain 10X (**c**). WDSCC showing intense MMP9 stain 20X.

Here, we present the results of protein expression of 3 biomarkers namely LAMC2 (Fig. 3), ECAD (Fig. 4) and MMP9 (Fig. 5) among the others which have been evaluated. The correlation of the IHC markers with various clinic-pathological parameters like age, sex, tobacco habits, alcohol habit, pathological stage, tumor grade, cervical node status, occult node status, perineural invasion status and type of growth was investigated.

### Panel of poor prognostic molecular markers in OTSCC validated by IHC for treatment outcome, pattern of failure, cervical lymph node involvement and locally advanced disease

As shown by the qPCR-based mRNA expression studies, the immunohistochemical analysis also confirmed that LAMC2 (χ^2^ = 9.45, *P* = 0.002), MMP9 (χ^2^ = 20.33, *P* < 0.001) and ECAD expression at ITF (χ^2^ = 18.05, *P* < 0.001) had a significant correlation with the treatment outcome. LAMC2 (χ^2^ = 12.97, *P* = 0.024) and MMP9 expression (χ^2^ = 20.5, *P* = 0.001), also correlated with the pattern of failure. Altered ECAD expression at ITF and MMP9 overexpression denoted nodal positivity and poor outcome in OTSCC. The pattern of ECAD expression at ITF (χ^2^ = 6.0, *P* = 0.01) and over-expression of MMP9 (χ^2^ = 7.09, *P* = 0.008) correlated with the cervical node status. MMP9 also correlated with the locally advanced stage of tumor (χ^2^ = 7.09, *P* = 0.008) with higher MMP9 expression seen in 84.4% of all locally advanced tumors.

### Clinico-pathological factors and molecular markers influencing tumor recurrence and as predictors of death analysed by univariate and multivariate analyses

The DFS median was 24 months with a range of 0 to 54 months while the median for OS was 30 months with a range of 4 to 74 months. Univariate and multivariate analyses were performed to identify the risk factors for recurrence of disease and death due to cancer using the Cox proportional hazard regression model (Table 5). Kaplan Meier survival analyses were also done to identify the significant predictors of DFS and OS.

**Table 5:** Univariate survival analysis for disease-free and overall survival in all patients.

Variables	Disease-free survival			Overall survival		
	Hazard ratio (95% C.I.)	*P* value	Events	Hazard ratio (95% C.I.)	*P* value	Events
Age	0.91 (0.45–1.84)	0.8	9 (47.4)	1.07 (0.47–2.43)	0.87	7 (36.8)
Gender	0.73 (0.35–1.51)	0.4	10 (43.5)	0.94 (0.43–2.05)	0.87	9 (39.1)
Tobacco chewing	**2.29 (1.2–4.34)**	**0.01**	**35 (61.4)**	**2.45 (1.16–5.21)**	**0.02**	**30 (53.6)**
Tobacco smoking	1.39 (0.77–2.5)	0.28	19 (63.3)	1.74 (0.91–3.35)	0.09	17(56.7)
Alcohol	**1.85 (1.04–3.29)**	**0.04**	**24 (61.5)**	**2.36 (1.23–4.55)**	**0.01**	**22 (57.9)**
Habits	**1.46 (1–2.13)**	**0.05**	**22 (55)**	**1.78 (1.12–2.84)**	**0.01**	**24 (58.5)**
Node status	**2.6 (1.39–4.88)**	**0.003**	**35 (67.3)**	**2.67 (1.29–5.52)**	**0.008**	**29 (58)**
Stage	**2.16 (1.18–3.96)**	**0.01**	**34 (66.7)**	**2.45 (1.21–4.96)**	**0.01**	**29 (59.2)**
Grade	1.18 (0.77–1.82)	0.45	10 (66.7)	**1.68 (1.02–2.78)**	**0.04**	**10 (66.7)**
PNI	0.99 (0.44–2.22)	0.99	7 (46.7)	0.85 (0.33–2.17)	0.73	5 (38.5)
Occult node status	**4.27 (1.53–11.91)**	**0.005**	**8 (53.3)**	**5.19 (1.46–18.46)**	**0.01**	**6 (46.2)**
LAMC2	**2.69 (1.38–5.22)**	**0.004**	**33 (66)**	**2.91 (1.36–6.21)**	**0.006**	**27 (54)**
ECAD at ITF	**3.19 (1.64–6.23)**	**0.001**	**31 (75.6)**	**3.11 (1.48–6.51)**	**0.003**	**24 (58.5)**
MMP9	**5.39 (1.88–15.48)**	**0.002**	**31 (83.8)**	**3.09 (1.07–8.9)**	**0.04**	**25 (67.6)**

Factors denoting increased hazard of death and recurrence in OTSCC; Numbers in brackets denote percentages. The numbers in bold denote values that are of statistical significance.

**Figure 6:**
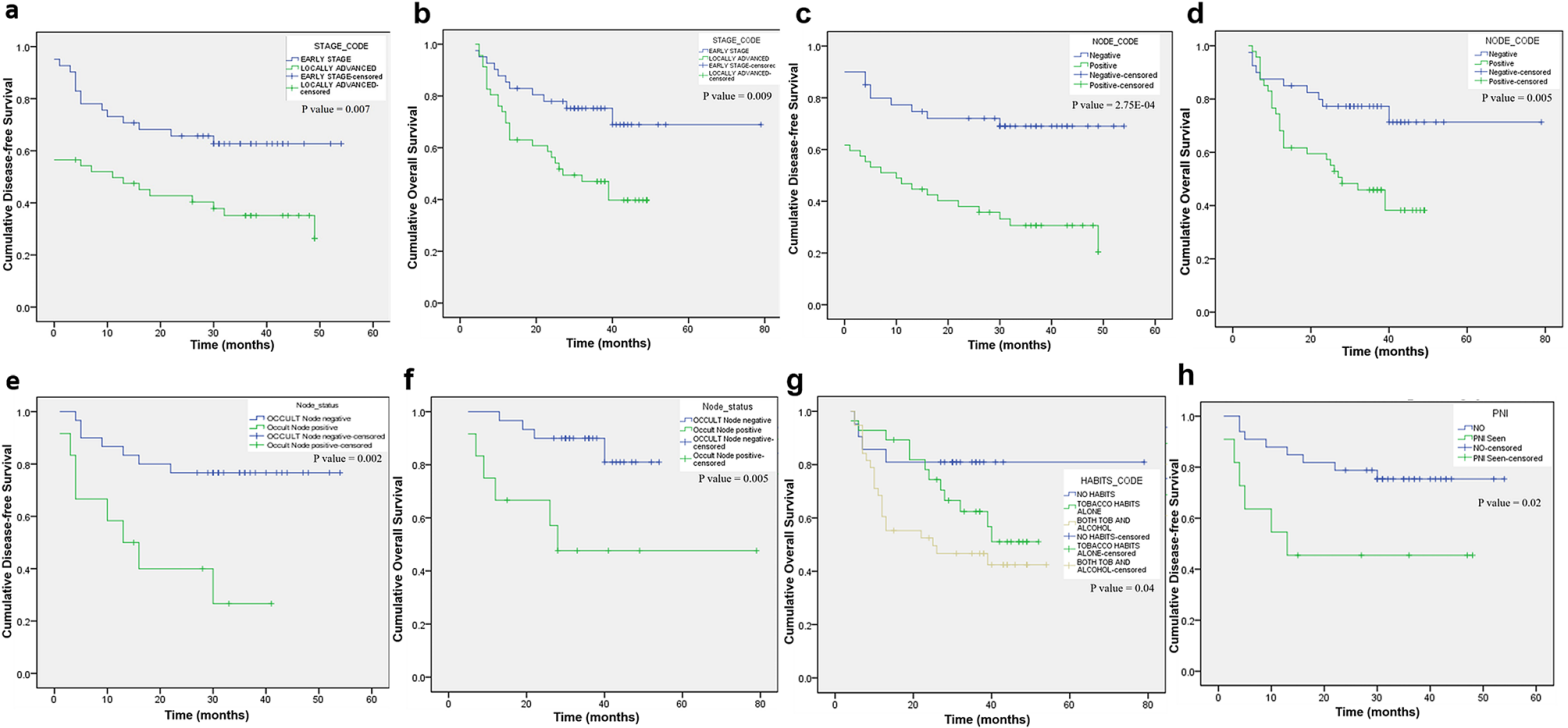
Kaplan–Meier survival analysis of clinicopathological variables in OTSCC patients (**a**). Disease free survival by clinical stage (**b**). Overall survival by clinical stage (**c**). Disease free survival by neck node status (**d**). Overall survival by neck node status (**e**). Disease free survival by occult node status (**f**). Overall survival by occult node status (**g**). Overall survival by habits status (**h**). Disease free survival by Perineural invasion in early stage OTSCC treated by surgery. *P* values correspond to the log-rank test comparing the survival curves.

Among all the patients, the classical prognostic factors like habits—tobacco chewing (*P* = 0.01), alcohol (*P* = 0.04), clinical factors like node status (*P* = 0.003), stage (*P* = 0.01) and occult node status (*P* = 0.005) were found to be significant predictors of disease recurrence in OTSCC. Tobacco chewing, positive nodal status and advanced stage of tumor increased the hazard of tumor recurrence by more than two-fold while a positive occult node increased the risk of recurrence by 4.27-fold. The same clinico-pathological parameters were also significant predictors of death. Tobacco chewing (*P* = 0.02) and alcohol (*P* = 0.01) were associated with an increased hazard of death as seen by the HR of more than 2. Similarly, positive cervical node status (*P* = 0.008), advanced clinical stage (*P* = 0.01) and grade (*P* = 0.04) of the tumor were also significant predictors of death. Occult node positivity increased the risk of death by 5.19-fold and was the most significant clinic-pathological parameter among all factors.  Kaplan–Meier survival analysis also confirmed the parameters clinical stage, node, presence of perineural invasion and occult node status to be predictors of disease recurrence and death (Fig. 6) with the presence of tobacco habits identified as an additional predictor of death.

**Figure 7:**
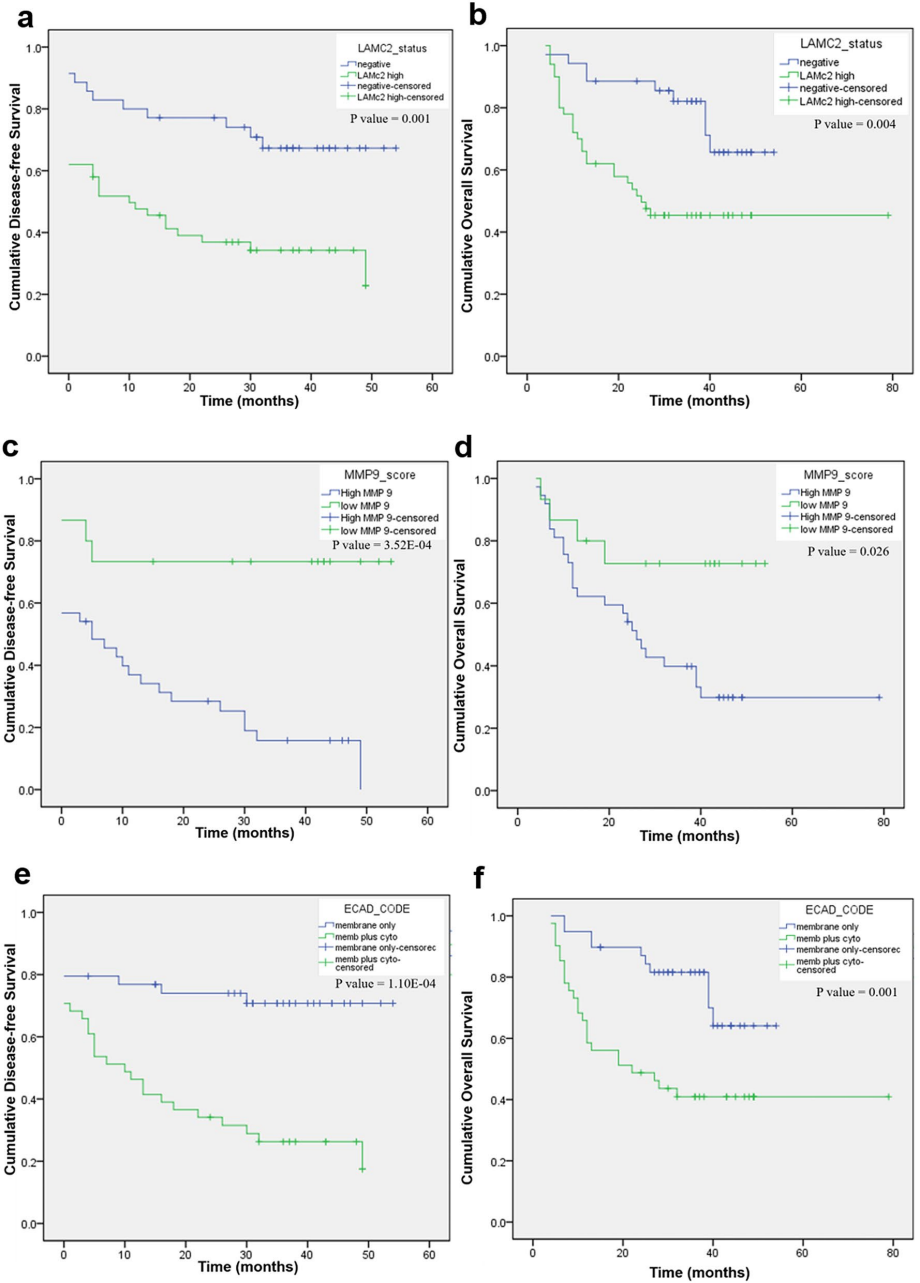
Kaplan–Meier survival analysis of molecular markers in OTSCC patients (**a**). Disease free survival by LAMC2 immunoexpression (**b**). Overall survival by LAMC2 immunoexpression (**c**). Disease free survival by MMP9 immunoexpression (**d**). Overall survival by MMP9 immunoexpression (**e**). Disease free survival by ECAD immunoexpression (**f**). Overall survival ECAD immunoexpression. *P* values correspond to the log-rank test comparing the survival curves.

Among the molecular markers, high LAMC2 expression (Fig. 7a,b) was associated with an increased risk of recurrence (*P* = 0.004) and an increased risk of death (*P* = 0.006). Loss of membrane positivity of E-cadherin was associated with an increased hazard of disease-recurrence as seen by the increased HR of 3.19 for DFS (*P* = 0.001), when compared to no E-cadherin expression. It was also a significant predictor of death (*P* = 0.003) with a HR of 3.11. High MMP9 expression was associated with an increased risk of both disease recurrence (*P* = 0.002) and death (*P* = 0.004). These results were also mirrored by the Kaplan–Meier survival analysis of factors predicting DFS and OS (Fig. 7c,d) (Supplementary Table S9). Among the molecular markers, ECAD expression pattern at the ITF was the most significant predictor of both disease recurrence (*P* = 0.002) and death (*P* = 0.03) with an increased HR of 25.39 and 10.81 respectively. These results were confirmed by the Kaplan–Meier survival analysis (Fig. 7e,f).

## Discussion

OTSCC is an aggressive cancer with high invasion and metastatic properties. Lymph node metastasis is the single most important prognostic indicator, however, in early stages, the lymph node metastasis is occult without clinical signs. Though, elective neck dissection is recommended for early stages, majority of the patients may not benefit substantially due to the surgery related morbidities. Biomarkers are therefore important in OTSCC and more importantly in early stages to prevent mortality and in identifying the high-risk patients. There is a need for reliable tests to detect aggressive early stage OTSCC to provide adequate treatment for these patients.

The findings of our current transcriptomic sequencing study were in complete agreement to our published meta-analysis study on OTSCC^22^. This study shows that role of tumor microenvironment in OTSCC with a number of extracellular matrix (ECM) components playing a crucial role in patient prognosis. Based on the data obtained from RNA-seq, the top up regulated genes included members of Matrix metalloproteinases and Melanoma associated gene families. The Gene Ontology terms enriched among the DEGs included inflammatory response, ECM organization, cell adhesion, collagen catabolic process and metalloendopeptidase activity. Studies have shown that biomarkers of stromal microenvironment might have a greater impact on prognosis compared to biomarkers related to tumor cells^23^. We found ECM receptor interaction and Focal adhesion as the most significant deregulated pathways in OTSCC. However, biomarkers related to tumor microenvironment have not been widely studied except activin A^24^. Degradation of the ECM that expediates the movement of tumor cells to surrounding tissue, is one of the important hallmarks of cancer progression and is facilitated by collagenases. Focal adhesion kinase is a tyrosine kinase that mediates signaling by the integrin family of cell surface receptors for ECM, leading to tumor cell migration and progression^25^. The module analysis additionally identified Neuroactive ligand–receptor interaction pathway, which is implicated in OTSCC^26^ and other types of cancers, like breast^27^ and bladder^28^ to be dysregulated in OTSCC. Collectively, these results complement each other and reinforce the pivotal role played by ECM remodeling and stromal microenvironment in OTSCC which in turn clarifies the aggressive nature of the disease and its propensity to metastasize.

The hub genes in a network are expected to be key drivers influencing the normal functioning of a cell, owing to the central role played by them in the network with multiple interactions^29^. APP was identified as the most significant hub gene in both transcriptomic and meta-analysis studies^23^. APP, a type-I integral membrane protein, has shown higher expression levels in OSCC and is known to be associated with poor prognosis^30^. Secretory proteins assume a lot of importance in the context of a high-effect local disease like oral cancer owing to its potency to be detected in saliva. With more than 500 of the DEGs in the current study being secreted proteins, patient-based saliva proteomics could be an engaging approach to biomarker discovery in oral cancer^31^. SPOCK1 was found to be up-regulated in OTSCC but its mode of action and its role in OSCC has not been explored and merits an exhaustive analysis. SPOCK1 has the potential to act as a prognostic marker and the added advantage of it being a secretory protein, offers the possibility of non-invasive detection.

While surgery is the accepted first line of treatment in OTSCC, there exists several views over the clinical management of node negative patients with suggestions ranging from observation to elective neck dissection^32–35^. However there has been no single, decisive prognostic/predictive factor for a positive occult node^32^ and clinical staging is insufficient and often underestimates the extent of occult node positivity^36^. These shortcomings have called for an exigent need to identify molecular markers to predict occult tumor burden and our study has attempted to answer it.

Our cohort comprised of stage I to stage IV cancers and was representative of the entire spectrum of OTSCC. Out of the 26 markers evaluated, 19 of the tested biomarkers were able to significantly differentiate tumor from normal samples. We report 12 genes namely, LAMC2, VIM, HIF1A, TWIST2, SOX2, TNC, PDPN, MMP9, DSG2, PLAU, FOXM1 and MYO1B found to be relevant clinical markers that can be easily tested in OTSCC tumors to indicate prognosis, cervical and occult node positivity and outcome. We have derived a panel of molecular markers significantly predicting recurrence of the early stage OTSCC based on the gene expression levels of 6 genes namely, MMP9, LAMC2, DSG2, PLAU, FOXM1 and MYO1B.

Several studies including our previous study have shown that members of MMP family are involved in OTSCC with very significant upregulation^23,37–39^. Additional studies by Zhou et al., showed up-regulation of the MMP9 gene to be associated with advanced OTSCC and having a predictive value for the identification of lymph node metastasis^40^. These evidences along with our findings emphasize the importance of evaluating MMP9 in OTSCC.

LAMC2 was identified as a relevant gene and an important indicator of poor prognosis and cancer progression in OTSCC^41,42^ with its overexpression indicating an advanced stage and positive cervical node status. Our results are in confirmation of earlier studies identifying LAMC2 as a prognostic factor in early stage and advanced stage OTSCC tumors^43^. Interestingly, in our cohort, the tumor area facing the stroma had a higher expression of LAMC2. Most of the extracellular spaces of the tumor areas showed accumulation of LAMC2 and expression of LAMC2 at tumor stroma interfaces indicates invasion and cancer progression^44^.

Other relevant genes were DSG2, and PLAU. DSG2 is an adhesion molecule and a known epithelial EMT marker overexpressed and is also known for bad prognosis marker in hepatocellular carcinoma^45^ and gastric carcinoma^46^. Our observation of PLAU as a marker of relapse in OTSCC is in line with prior study in OSCC demonstrating the role of PLAU as a strong independent prognostic factor of DFS^47^. Baker et al., have previously reported the over-expression of PLAU and its correlation to aggression of tumors in OSCC^48^.

Several studies have implicated FOXM1 to play a major role in chemo-resistance in gastric cancer^49,50^, glioblastoma^51^, non-small-cell lung cancer^52^ and most recently, also in oral cancer where it is proven to predict poor patient survival^53^. This is similar to our findings where FOXM1 overexpression is associated with relapse in OTSCC patients. MYO1B, a motor protein involve in cellular motility^54^, is known to be overexpressed in HNSCC^55^ and is found to play a pivotal role in lymph node metastasis by way of augmenting cancer cell motility^56^. SOX2 is a transcription factor and stem cell marker, known to be expressed in several cancers, playing a major role in cell proliferation, metastasis^57^, tumor drug resistance^58^ and is hence a valuable therapeutic target^59^. SOX2 maintains the stemness of CSC, ultimately resulting in cancer relapse and resistance to treatment^60^. Liu et al. demonstrated the involvement of SOX2 in promoting tumor aggressiveness and EMT in OTSCC^61^. In this scenario, our finding of SOX2 to be a marker of failure in OTSCC cases treated by radiotherapy is significant.

We identified TNC and PDPN to be significant markers of occult node metastasis. TNC, a protein of the extracellular matrix with important functions in angiogenesis and tumor cell proliferation^62^, has been established as a diagnostic marker and potential cancer-associated fibroblast marker in breast ductal cancer^63^ and cervical cancer^64^ and a prognostic marker in early stage OTSCC^65^. The functions of TNC and its role as a cancer stem cell (CSC) marker^66^ makes it an ideal marker of occult node positivity as found in our study. Another important marker of occult node positivity and poor outcome identified in radiation-treated locally advanced OTSCC was PDPN. PDPN is a trans-membrane receptor glycoprotein that increases clonal cell capacity, EMT, invasion and metastasis^67^, playing a pivotal role in several cancers. Preclinical studies have identified PDPN as a therapeutic target to combat several cancers including oral cancer^68–71^ and nasopharyngeal cancer^72^. These findings corroborate our identification of PDPN as an occult node and poor prognosis predictor.

Our cohort had about 26.9% of occult positive cases that falls within the range of previous studies reported^34^. In another study on OTSCCs from our centre, the incidence of occult metastasis was 32.69%^73^. Currently, sentinel Lymph node biopsy has been suggested as a minimally invasive technique for nodal staging to reduce the needless morbidity of nearly 70–75% of pathologically node negative patients undergoing elective neck dissection. Since, sentinel node is generally believed to be the first lymph node or lymph nodes group receiving lymphatic drainage from the primary tumor, if this node is metastasis negative, the non-sentinel nodes in the neighboring regional basins are also deemed to be negative of metastases. Currently, there are no molecular markers that can identify metastasis in lymphatic basins, and we suggest that TNC and PDPN expression can be evaluated in a larger series of cases to identify patients at risk of occult micrometastasis.

Survival analyses of the clinicopathological factors predicting recurrence and death identified clinical stage, cervical node, occult node and tobacco habits as the classic prognostic factors in tongue cancer^74,75^. Our validations based on protein expression predicting risk of disease relapse and death in OTSCC patients confirmed the expression of LAMC2, MMP9 and ECAD at ITF as the important prognostic indicators. Thus, among our panel of markers, MMP9, LAMC2, VIM, DSG2 and TNC are well-known EMT markers, emphasizing the importance of EMT in OTSCC.

In the exclusive cohort of early stage patients treated by surgery, cervical node and peri-neural invasion significantly predicted failure in early stage OTSCC as reported earlier^76,77^. Loss of membrane ECAD at ITF predicted both risk of recurrence and death. Among the locally advanced cases treated by radiation, LAMC2, MMP9 and POSTN were the predictors of disease relapse while node, LAMC2 and POSTN were the prognostic factors predicting death.

Murthy et al. reported node to be an important prognostic factor in oral cancer patients treated by radiotherapy and went on to state that among the different oral subsites, OTSCC patients treated by radiotherapy perform poorly^78^. Our locally advanced tumors were treated by radiation and POSTN turned out to be an important prognostic factor in these tumors. High levels of POSTN are found to be associated with aggressive tumor behaviour, tumor progression, resistance to treatment and hence, bad prognosis^79–81^. Although Choi et al. have identified POSTN to be a diagnostic marker in OSCC by tissue microarray^82^ and its role in OSCC tumorigenesis is well established^83,84^, its prognostic role in OTSCC has not been reported earlier.

Although many earlier studies have reported diagnostic biomarkers based on mRNA expression in OSCC^85^, very few have derived meaningful mRNA-based prognostic markers^86^, more so in OTSCC. Using a panel of markers has superior discriminatory power over using individual candidate genes and hence, our panel of biomarkers with further validations in a larger study, may have better utility in the clinical setting to indicate the risk of recurrencee in OTSCC.

## Conclusion

OTSCC transcriptome sequencing upholds prior findings on the pivotal role of ECM degradation in OTSCC tumorigenesis and progression. Our cohort study addressed the most pressing issue of OTSCC management, namely, occult node metastasis prediction with 2 molecular markers and identified a panel of 6 molecular markers that can be used to differentiate the early stage tumors at higher risk of recurrence. Identification of novel biomarkers for early detection, prediction of response, and for use as treatment targets is of utmost importance to increase survival in OTSCC and the current study has fulfilled that. With further validation in a larger cohort, preferably, a multi-centric one, the panel of prognostic markers ascertained in our study can be used routinely to make important clinical decisions with respect to treatment modalities.

## Methods

### Patient materials

The Ethical approval number of the study was CIWIA/Protocol 1/HNCOG 2014, namely Cancer Institute Womens India Association/ Protocol 1 /Head and Neck Oncology Group 2014). All research involving human participants had been approved by the authors’ Institutional Review Board (IRB) and all clinical investigations had been conducted according to the principles expressed in the Declaration of Helsinki. A written informed consent was obtained from all the participants and the content of the informed consent was approved by the Institutional Research Board.Prospective primary tongue cancer samples (n = 100), both punch biopsy and tissue samples from surgical specimens with corresponding apparently uninvolved adjacent tissue were collected from the patients presenting with OTSCC between the years 2014 and 2016 to the Head and Neck Oncology Clinic, Cancer Institute (WIA), Chennai, India. Collected samples were immediately immersed in RNA*later* and stored in − 80 °C till further use. The patients were treated as per the NCCN guidelines. Variables recorded and evaluated for the study included age, sex, site, size of the tumor, clinical stage, histological grade, tobacco habits, alcohol habits, comorbids like Diabetes mellitus and Hypertension, node status, perineural invasion and occult node status.

### Transcription sequencing

Transcriptome libraries for sequencing were constructed using 0.5 μg of total RNA as per manufacturer’s instructions (TruSeq RNA Sample Preparation Kit, Illumina) and was analysed as described previously^87^. Briefly, the libraries were multiplexed and sequenced on HiSeq 2500 to obtain on average ~ 108 million single end 95 bp reads per sample. RNA-seq reads were mapped to the human genome version NCBI GRCh37 using GSNAP. Only uniquely mapped reads were considered for downstream analysis. Gene models were based on RefSeq transcripts, NCBI and ENSEMBL gene annotations. Differential gene expression was performed with the R/Bioconductor package DESeq2. For clustering, count data were transformed by variance stabilization and genes were centered to have mean zero. Clustering was performed using 1-Pearson correlation as distance metric and average linkage. Heat maps were generated using the R/Bioconductor package NMF. Fold change was calculated based on comparing the average of tumor samples with the average of normal samples. The relative abundance metric parameter FPKM (Fragments Per Kilobase of exon per Million reads sequenced) was used to represent the value of gene expression. To detect and filter DEGs, we applied the Student’s *t* test (*P* values), and the FDR correction (*q*-values).

### Functional analyses

Functional interpretation of the DEGs was performed with Database for Annotation, Visualization and Integrated Discovery (DAVID, Version 6.8)^88^, a web-based tool for Gene Ontology (GO) and Kyoto encyclopedia of genes and genomes (KEGG) pathway enrichment analyses^89^. A Benjamini-corrected *P* value less than 0.05 was used to identify a statistically significant analysis. The hub proteins were identified using the Cytoscape v3.6.0. plugin called ‘Network Analyzer’^90^. The hub proteins were evaluated by analyzing the highest closeness centrality (CC), betweenness centrality (BC), and the node degree. Genes with degree ≥ 10 were defined as hub genes in the present study. In addition, the Molecular Complex Detection (MCODE) plugin of Cytoscape software was also employed to identify functionally related and highly interconnected clusters from the PPI network with a degree cutoff of 2, node score cutoff of 0.3, k-core of 4, and maximum depth of 100 (http://baderlab.org/Software/MCODE)^91^. Significant modules were identified with MCODE score ≥ 4 and nodes ≥ 6. Subsequently, based on modules selected from the PPI network, functional enrichment analysis was performed using DAVID with the criterion of *P* < 0.05. The protein–protein interactions PPI was visualised using Search Tool for the Retrieval of Interacting Genes (STRING) online database (v10.5) (www.string-db.org) for network construction^92^.

### qPCR

Total RNA was extracted from the tissue samples, homogenising them in liquid nitrogen, using RNeasy mini kit (Qiagen, Hilden, Germany) using standard manufacturer protocol. Total RNA (2 µg) was converted to cDNA using High capacity cDNA reverse transcription kit (Applied Biosystems, California, U.S.A.) as per instructions of the manufacturer and stored at − 40 °C.Primers used for the study are listed in Supplementary Table S7. The quantitative real-time RT-PCR was performed using FastStart Universal SYBR Green Master (Rox) (Roche, Basel, Switzerland) in a 20 µl reaction mix according to the manufacturer's instructions on a 7500 Real Time PCR System (Applied Biosystems, California, U.S.A.). The thermal cycling conditions used were as mentioned before^23^. Triplicates were performed for each gene and average expression value was computed for subsequent analysis. The relative expression level of the genes was calculated using the 2^−∆∆Ct^ method. We defined the cut-off value for over-expression of genes based on the median log RQ values for each of the marker. Patients with pathological stages I and II disease were grouped as ‘early stage’ tumors and patients with pathological stages III and IV were grouped as ‘advanced stage’ tumors. We used *t* test to identify the markers whose expression varied most among the staging, node positive and outcome subgroups.

### Immunohistochemistry

The IHC methodology is mentioned previously^23^. The IHC detection of MMP9, LAMC2, ECAD, was performed on five-micron sections of FFPE tissues. The sections were deparaffinised in xylene, rehydrated in absolute ethanol and endogenous peroxidase activity was blocked by incubation in 0.3% hydrogen peroxide in Phosphate-buffered Saline (PBS) for 30 min. The sections were then subjected to heat-induced epitope retrieval according to the conditions listed in Supplementary Table S8. Briefly, the sections were blocked in 2% Bovine Serum Albumin (BSA) for 30 min and then incubated with primary monoclonal antibody overnight at 4 °C. The marker expression was observed using the SuperSensitive Polymer-HRP IHC Detection System (BioGenex Laboratories, San Ramon, CA) as per the instructions of the manufacturer. Primary antibody was replaced with 2% BSA in negative control and suitable positive control specific to each of the markers was used. Immunostaining of the sections was reviewed along with corresponding Haematoxylin and Eosin stained sections.

### Statistical analysis

Overall Survival (OS) was calculated as time in months from the date of primary treatment to the date of death due to the disease. Disease free survival (DFS) was calculated as time in months from the date of primary treatment to the date of objective tumor recurrence (local, nodal, locoregional, and distant metastases). The samples were blinded with respect to clinical data during the molecular testing. All statistical analysis was done using SPSS, version 16 (IBM).

## Supplementary information

Supplementary Information 1.

Supplementary Information 2.
