# Supplementary material for: Deregulation of extracellular matrix modeling with molecular prognostic markers revealed by transcriptome sequencing and validations in Oral Tongue squamous cell carcinoma
Source: Sci Rep. 2021 Jan 8;11:250. doi: 10.1038/s41598-020-78624-4 (PMC7794513; doi:10.1038/s41598-020-78624-4)
Supplement: Supplementary file 1 — Supplementary Information 1. [file 41598_2020_78624_MOESM1_ESM.doc]

**Supplementary Data 1**

Deregulation of extracellular matrix modeling with molecular prognostic markers revealed by transcriptome sequencing and validations in Oral Tongue squamous cell carcinoma

**Authors**

Soundara Viveka Thangaraj^＊^, Vidyarani Shyamsundar^＊^, Arvind Krishnamurthy ^#^, Vijayalakshmi Ramshankar^＊^

**Supplementary Figures**

Supplementary Figure S1. **Heatmap representing the expression of top 100 DEG in OTSCC obtained by transcriptome sequencing**


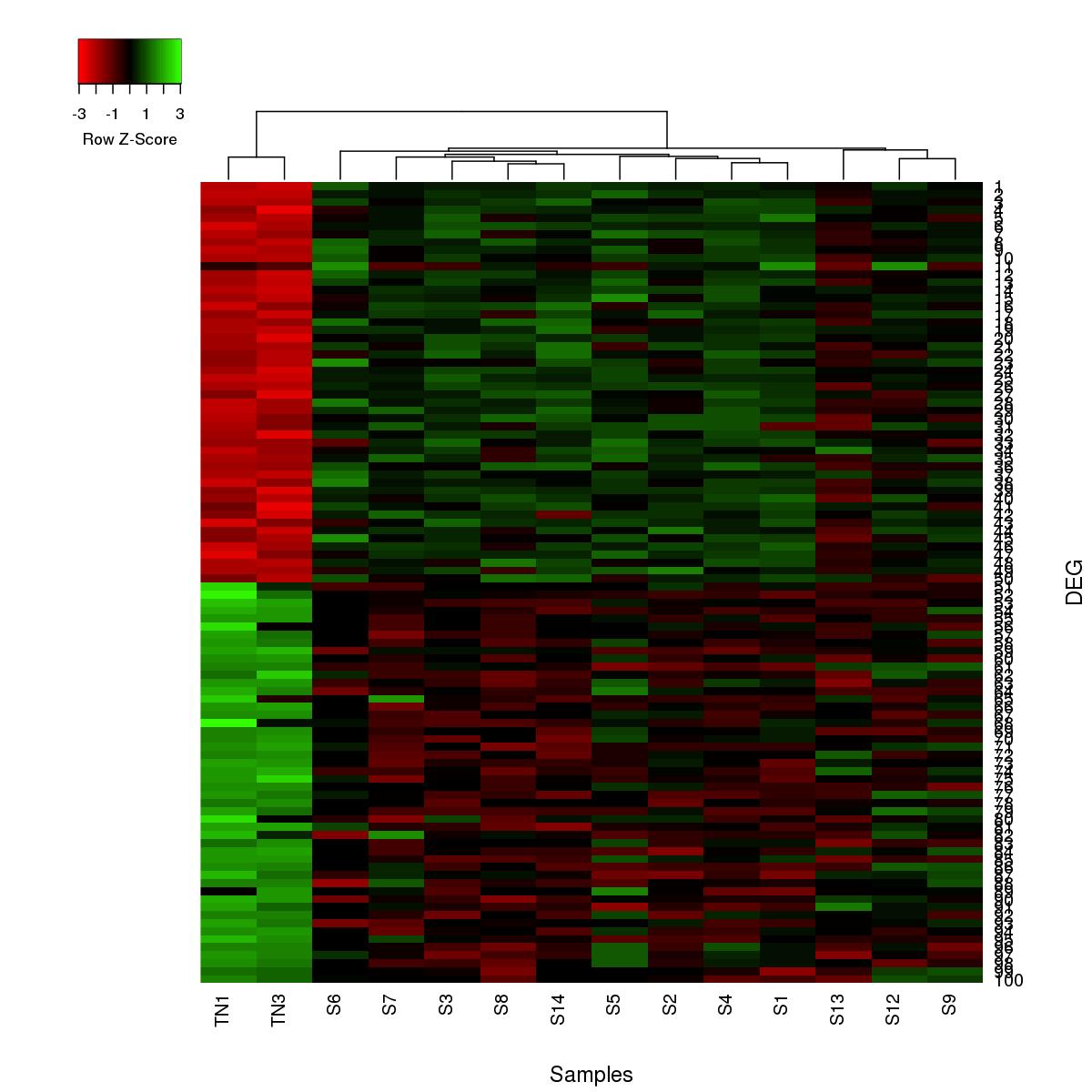


Supplementary Figure S2. **GO enrichment and KEGG Pathway analysis of DEG in OTSCC** Topmost enriched GO terms for differentially expressed genes. A. GO terms of Biological process B. GO terms of Molecular function and C. GO terms of Cellular component. D. Pathway analysis based on the KEGG database^89^. The bar plot shows the enrichment scores (-log (P value)) of the significant enrichment GO terms / pathway.


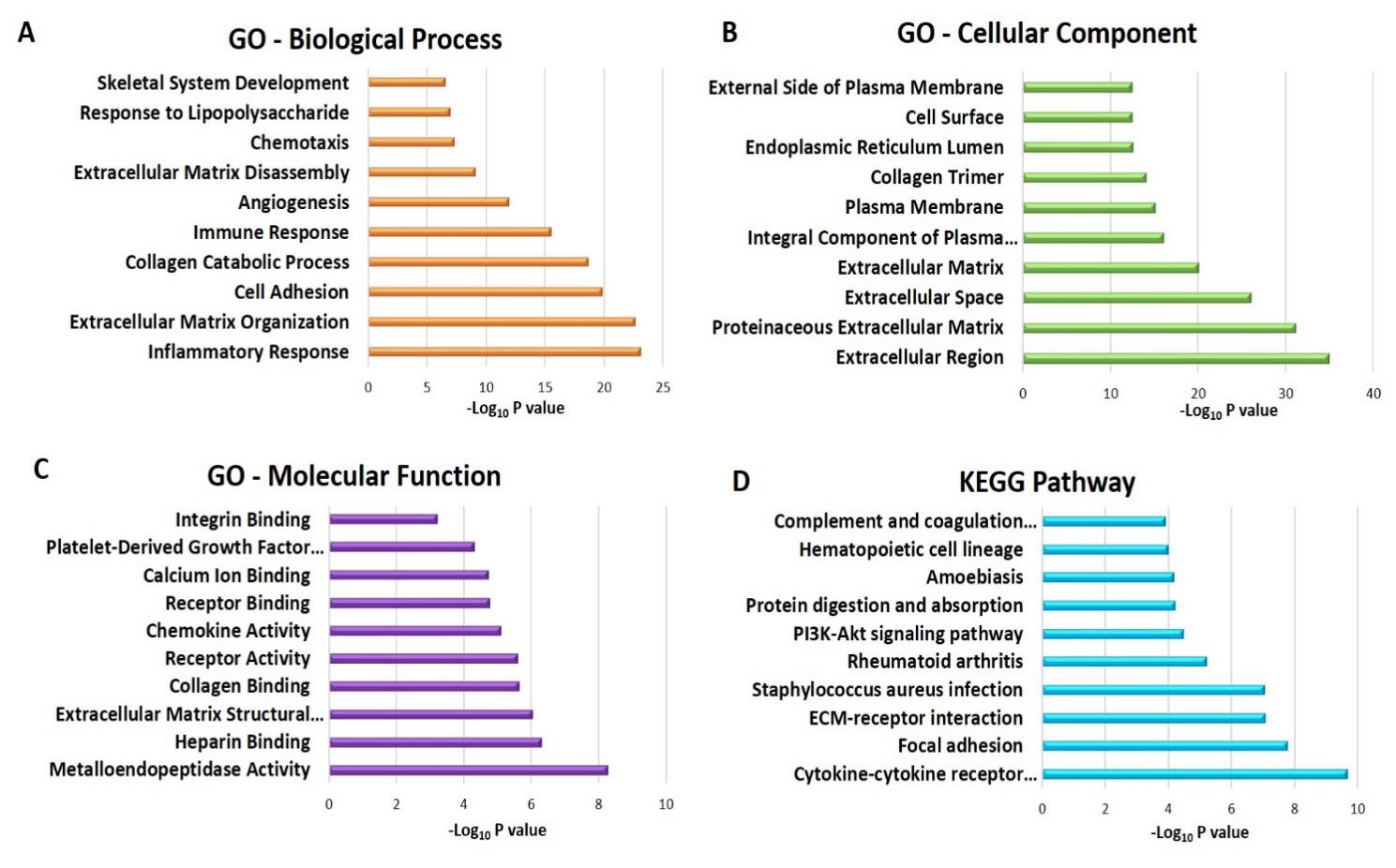


Supplementary Figure S3. **STRING- based** **Integrated subnetwork of the top most significant modules of OTSCC-PPI network**. A. Module 1 with MCODE score 30 B. Module 2 with MCODE score 21.37. The nodes represent proteins, each edge represents an interaction and the line thickness indicates the confidence level^92^.


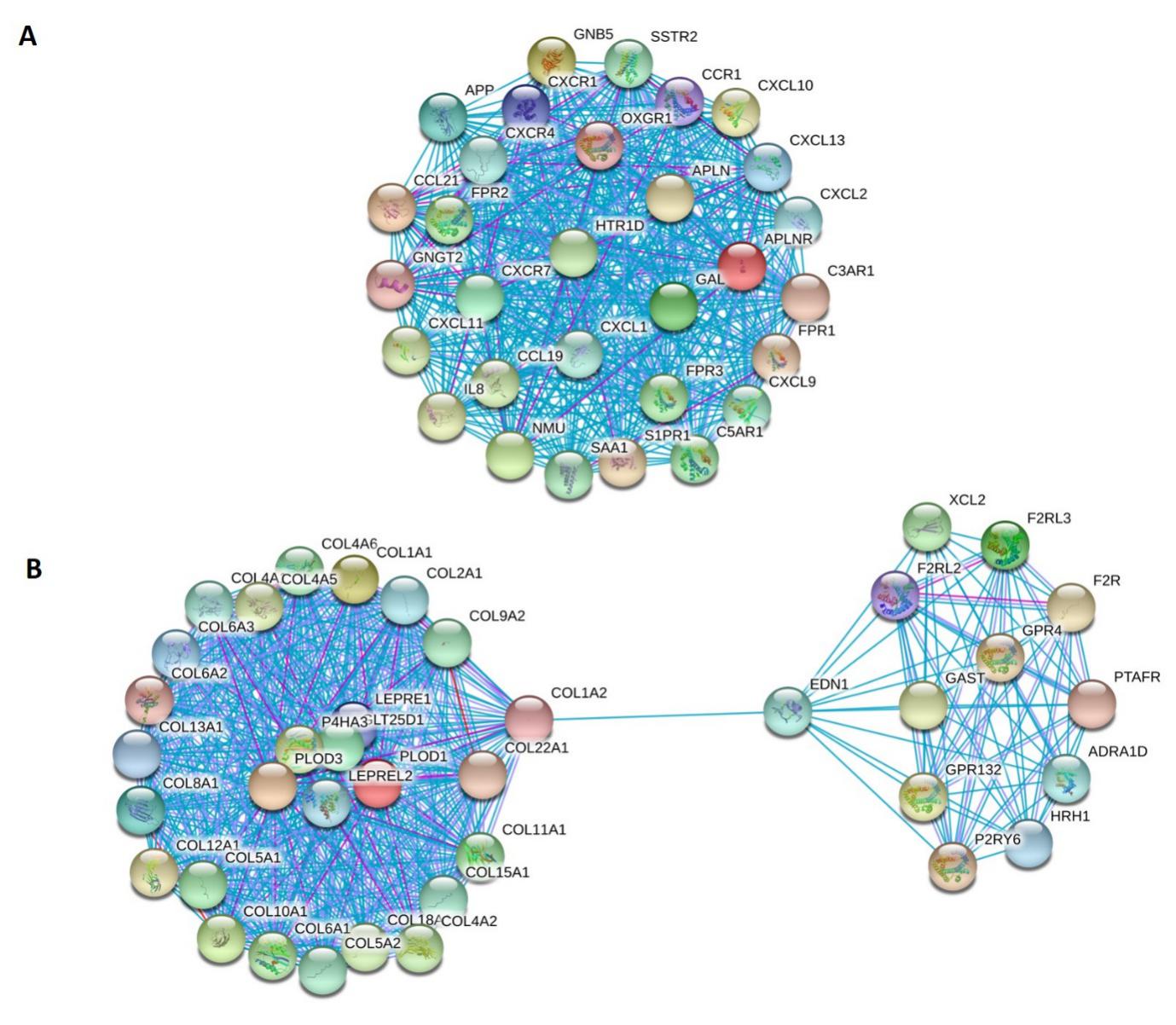


Supplementary Figure S4. **Molecular markers to differentiate clinical stage in OTSCC:** Boxplot showing the expression of genes a. LAMC2 (P value = 0.02) b. HIF1A (P value = 0.01) c. TWIST2 (P value = 0.03) d. SOX2 (P value = 0.02) e. VIM (P value = 0.02) among early stage and advance stage OTSCC.


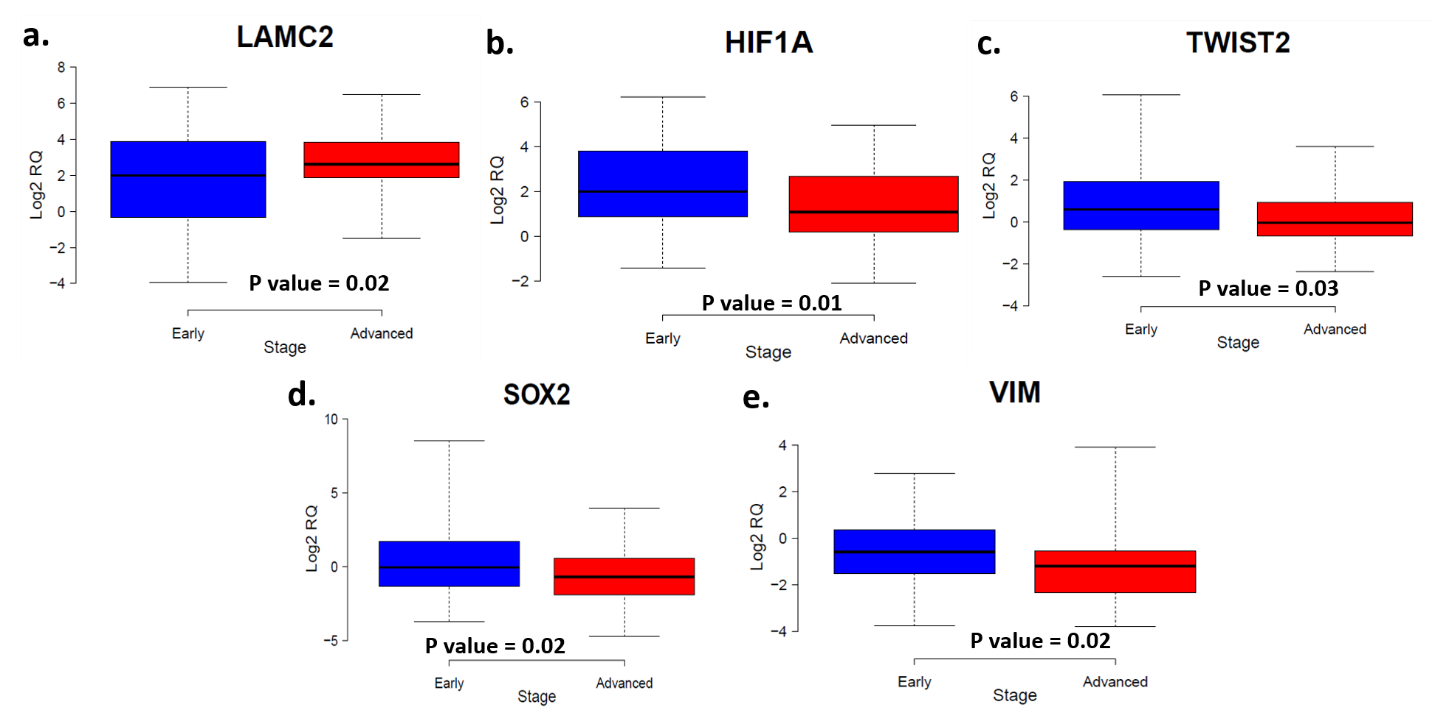


**Supplementary Tables**

**Table S1: Clinico**-pathological features of the OTSCC study patients (n=12) used for transcriptome sequencing

| **Patient** | **Age (yrs)** | **Sex** | **Site** | **Clinical Stage** | **Grade** | **Tobacco Habits** | **Alcohol Habits** | **Node status** |
| --- | --- | --- | --- | --- | --- | --- | --- | --- |
| S1 | 56 | Female | Lateral Border | Stage 4 | PDSCC | Chewing | Nil | Positive |
| S2 | 69 | Male | Lateral Border | Stage 1 | MDSCC | Never | Nil | Negative |
| S3 | 49 | Female | Lateral Border | Stage 3 | WDSCC | Never | Nil | Positive |
| S4 | 40 | Male | Lateral Border | Stage 3 | MDSCC | Chewing | Nil | Positive |
| S5 | 55 | Female | Lateral Border | Stage 3 | MDSCC | Never | Nil | Positive |
| S6 | 65 | Male | Ventral Aspect | Stage 2 | MDSCC | Chewing | Nil | Positive |
| S7 | 66 | Male | Lateral Border | Stage 3 | WDSCC | Chewing | Nil | Positive |
| S8 | 44 | Male | Lateral Border | Stage 4 | MDSCC | Never | Nil | Positive |
| S9 | 61 | Male | Lateral Border | Stage 3 | MDSCC | Smoking | Yes | Positive |
| S10 | 50 | Female | Lateral Border | Stage 4 | MDSCC | Chewing | Nil | Positive |
| S11 | 55 | Male | Lateral Border | Stage 3 | PDSCC | Smoking | Yes | Positive |
| S12 | 45 | Male | Lateral Border | Stage 3 | MDSCC | Never | Nil | Positive |

Table S2: Differentially expressed genes in OTSCC identified by transcriptome sequencing, with cut-off fold change >10, P< 0.01 (Supplementary Data 2)

**Table S3:** Top 10 Hub genes in the overall protein-protein interaction network

| **Gene Name** | **Betweenness** | **Gene Name** | **Degree** |
| --- | --- | --- | --- |
| APP | 0.13 | APP | 73 |
| IL8 | 0.09 | GNGT2 | 66 |
| TGFB1 | 0.08 | GNB5 | 66 |
| GNGT2 | 0.07 | SAA1 | 51 |
| GNB5 | 0.07 | FPR2 | 51 |
| PDE6A | 0.07 | IL8 | 50 |
| HLA-DRB5 | 0.07 | NMU | 50 |
| LYN | 0.06 | GNAI2 | 45 |
| IL6 | 0.06 | S1PR1 | 43 |
| PTGS2 | 0.06 | CXCR4 | 43 |

**Table S4:** Module analysis using MCODE

| Cluster | Score (Density*#Nodes) | Nodes | Edges | Node IDs |
| --- | --- | --- | --- | --- |
| 1 | 30 | 30 | 435 | NMU, SSTR2, CXCR7, CXCR1, IL8, CXCL9, CXCR4, APLNR, C3AR1, APLN, C5AR1, CCR1, CXCL1, CXCL11, OXGR1, CXCL10, FPR1, S1PR1, FPR3, SAA1, FPR2, CXCL13, CCL21, CCL19, GAL, CXCL2, APP, GNB5, GNGT2, HTR1D |
| 2 | 21.37 | 39 | 406 | LEPRE1, F2R, HRH1, EDN1, COL1A2, ADRA1D, COL22A1, GPR132, COL10A1, GPR4, COL8A1, COL18A1, PTAFR, COL12A1, COL4A5, COL6A1, COL5A2, COL4A2, LEPREL2, COL4A1, P4HA3, COL13A1, COL15A1, COL1A1, COL9A2, COL2A1, PLOD1, PLOD3, F2RL3, GLT25D1, P2RY6, F2RL2, COL6A3, COL5A1, COL11A1, GAST, XCL2, COL4A6, COL6A2 |
| 3 | 15.37 | 20 | 146 | IRF1, HLA-DRB5, IRF7, HLA-C, IFITM3, GBP6, IFITM1, HLA-B, BST2, IFIT3, IFIT2, HLA-F, TRIM46, OAS2, FCGR1A, OASL, IFI35, IFI6, GBP5, ICAM1 |
| 4 | 15 | 15 | 105 | CCNF, RCHY1, CDC20, KLHL5, ASB11, ASB2, UBE2L6, ASB8, PJA1, SOCS1, FBXO40, CUL3, KBTBD13, CBLB, SOCS3 |
| 5 | 14.59 | 28 | 197 | ADAMTS2, F5, ADAMTSL2, THSD4, ADAMTS3, THSD1, A2M, THBS1, SPARC, ADAMTS4, PDGFB, TGFB3, ADAMTS18, ADAMTS15, SERPINE1, ISLR, THBS2, TIMP1, SERPINA1, VEGFC, ADAMTS9, FERMT3, ADAMTS7, TGFB1, SEMA5B, FAM3C, ADAMTS12, SRGN |
| 6 | 9 | 9 | 36 | ADCYAP1, HRH2, RAMP3, PTHLH, HTR7, ADM, GPR84, PTGIR, PTGER2 |
| 7 | 7.38 | 30 | 107 | LUM, MMP7, NCF1, NCF2, MUC16, MUC21, GALNT6, MUC12, KIF26B, MUC3A, MMP1, ST3GAL2, ST6GALNAC3, MMP13, ACAN, GALNTL4, ARFGAP1, CYBA, MUC13, KDELR3, MUCL1, MMP9, B3GNT7, GALNT10, KIF3C, B3GNT3, KIF21B, KIF19, MMP10, MMP3 |

Table S5: **Functional enrichment analysis of modules of PPI network**.

| **MODULE 1** | | | **MODULE 2** | | |
| --- | --- | --- | --- | --- | --- |
| **Identifier** | **Description** | **P value** | **Identifier** | **Description** | **P value** |
| GO Biological Process | | | GO Biological Process | | |
| GO:0006954 | Inflammatory response | 1.0E-17 | GO:0030574 | Collagen catabolic process | 1.3E-33 |
| GO:0006935 | Chemotaxis | 5.9E-17 | GO:0030198 | Extracellular matrix organization | 4.1E-22 |
| GO:0060326 | Cell chemotaxis | 8.8E-16 | GO:0030199 | Collagen fibril organization | 3.3E-10 |
| GO Cellular Component | | | GO Cellular Component | | |
| GO:0005615 | Extracellular space | 9.4E-05 | GO:0005788 | Endoplasmic reticulum lumen | 1.0E-31 |
| GO:0005887 | Integral component of plasma membrane | 8.1E-03 | GO:0005581 | Collagen trimer | 4.0E-29 |
| GO:0005886 | Plasma membrane | 1.3E-02 | GO:0005576 | Extracellular region | 5.3E-19 |
| GO Molecular Function | | | GO Molecular Function | | |
| GO:0008009 | Chemokine activity | 7.1E-11 | GO:0005201 | Extracellular matrix structural constituent | 4.9E-15 |
| GO:0048248 | CXCR3 chemokine receptor binding | 2.7E-06 | GO:0048407 | Platelet-derived growth factor binding | 4.9E-10 |
| GO:0004982 | N-formyl peptide receptor activity | 1.1E-03 | GO:0015057 | Thrombin receptor activity | 1.7E-03 |
| KEGG Pathway | |  | KEGG Pathway | | |
| hsa04062 | Chemokine signaling pathway | 1.8E-12 | hsa04974 | Protein digestion and absorption | 1.1E-27 |
| hsa04060 | Cytokine-cytokine receptor interaction | 1.0E-05 | hsa04512 | ECM-receptor interaction | 3.9E-14 |
| hsa04080 | Neuroactive ligand-receptor interaction | 4.1E-05 | hsa04510 | Focal adhesion | 1.4E-09 |

**Table S6:** Clinicopathological features of the prospective OTSCC Cohort

| **Clinico-pathological variable** | **Total (n=100)** | **Surgery Arm (n=52)** | **ConcurrentChemo-radiation Arm (n=48)** |
| --- | --- | --- | --- |
| **Age** |  |  |  |
| <45 | 39 | 19 (36.5) | 20 (41.66) |
| 46 to 65 | 48 | 23 (44.2) | 25 (52) |
| >65 | 13 | 10 (19.2) | 3 (6.25) |
| **Sex** |  |  |  |
| Male | 76 | 39 (75) | 37 (77) |
| Female | 24 | 13 (25) | 11 (22.9) |
| **Clinical Stage** |  |  |  |
| T1 | 14 | 14 (26.9) |  |
| T2 | 26 | 26 (50) |  |
| T3 | 20 | 12 (23) | 8 (16.66) |
| T4 | 40 |  | 40 (83.33) |
| **Grade** |  |  |  |
| WDSCC | 29 | 17 (32.6) | 12 (25) |
| MDSCC | 56 | 31 (59.6) | 25 (52) |
| PDSCC | 15 | 4 (7.69) | 11 (22.9) |
| **Tobacco Habits** | |  |  |
| Chewer | 39 | 14 (26.92) | 25 (52.0) |
| Smoker | 11 | 7 (13.46) | 4 (8.33) |
| Chewer and smoker | 19 | 6 (11.53) | 13 (27.08) |
| Non user | 31 | 24 (46.15) | 7 (14.58) |
| **Alcohol use** |  |  |  |
| Yes | 40 | 17 (32.6) | 23 (47.91) |
| No | 60 | 35 (67.3) | 25 (52) |
| **Comorbids** |  |  |  |
| Diabetes | 20 | 15 (28.8) | 5(10.41) |
| Hypertension | 15 | 4 (7.69) | 4 (8.33) |
| Nil | 72 | 33 (63.4) | 39 (81.25) |
| **Node** |  |  |  |
| Positive | 53 | 12 (23) | 41 (85.41) |
| Negative | 47 | 40 (76.92) | 7 (14.5) |
| **Occult node** | | |  |
| Positive | 15 | 15 (28.8) |  |
| Negative | 37 | 37 (67.3) |  |
| **Treatment Outcome** | |  |  |
| No Evidence of Disease | 49 | 38 (73) | 11 (22.9) |
| Failure | 50 | 14 (26.9) | 36 (75) |
| Lost to Follow-up | 1 | 0 | 1 (2.08) |
| **Survival** | |  |  |
| Alive | 68 | 44 (84.6) | 24 (50) |
| Dead | 31 | 8 (15.38) | 23 (47.91) |
| Lost to Follow-up | 1 | 0 | 1(2.08) |

**Table S7:** Primers used for Real-time quantitative PCR

| **Primer Name** | **Primer Sequence (5'- 3')** | **Accession** | **Product Size (bp)** | **Reference** |
| --- | --- | --- | --- | --- |
| ACTB_F | GAGCACAGAGCCTCGCCTTT | NM_001101.3 | 108 | S1 |
| ACTB_R | ACATGCCGGAGCCGTTGTC |  |  |  |
| MMP9_F | CTTTGACAGCGACAAGAAGTGG | NM_004994.2 | 111 | S2 |
| MMP9_R | GGCACTGAGGAATGATCTAAGC |  |  |  |
| SPP1_F | ACAGCCAGGACTCCATTGA | NM_001251830 | 241 | S3 |
| SPP1_R | TCAGGTCTGCGAAACTTCTTAG |  |  |  |
| FOXM1_F | GCAGGCTGCACTATCAACAA | NM_202002 | 154 | S4 |
| FOXM1_R | TCGAAGGCTCCTCAACCTTA |  |  |  |
| RBP1_F | CAACTGGCTCCAGTCACTCC | NM_001130992 | 80 | S5 |
| RBP1_R | CCTCGAAATTCTCGTTGACC |  |  |  |
| DSG2_F | ATCAATGCAACAGATGCAGATGA | NM_001943 | 174 | S6 |
| DSG2_R | TGTCAAAGTGTAGCTGCTGTGT |  |  |  |
| S100A7_F | AAATACACCGGACGTGATGG | NM_176823 | 139 | S7 |
| S100A7_R | TCTTGTCCTTTTTCTCAAAGACAGT |  |  |  |
| ECAD_F | TTCCTCCCAATACATCTCCC | NM_004360 | 142 | S8 |
| ECAD_R | TTGATTTTGTAGTCACCCACC |  |  |  |
| VIM_F | CTCTTCCAAACTTTTCCTCCC | NM_003380 | 134 | S8 |
| VIM_R | AGTTTCGTTGATAACCTGTCC |  |  |  |
| CDKN2A_F | GAAGGTCCCTCAGACATCCCC | NM_001195132 | 94 | S9 |
| CDKN2A_R | CCCTGTAGGACCTTCGGTGAC |  |  |  |
| CA9_F | CCTCAAGAACCCCAGAATAATGC | NM_001216 | 71 | S10 |
| CA9_R | CCTCCATAGCGCCAATGACT |  |  |  |
| HIF1α_F | CATAAAGTCTGCAACATGGAAGGT | NM_001530 | 148 | S11 |
| HIF1α_R | ATTTGATGGGTGAGGAATGGGTT |  |  |  |
| GLUT1_F | TTGGCTCCGGTATCGTCAAC | NM_006516.2 | 221 | S12 |
| GLUT1_R | GCCAGGACCCACTTCAAAGA |  |  |  |
| TWIST2_F | GAGCGACGAGATGGACAATAAGA | NM_001271893 | 84 | S13 |
| TWIST2_R | ATGCGCCACACGGAGAA |  |  |  |
| SOX2_F | GGGAAATGGGAGGGGTGCAAAAGAGG | NM_003106 | 151 | S1 |
| SOX2_R | TTGCGTGAGTGTGGATGGGATTGGTG |  |  |  |
| OCT4_F | GACAGGGGGAGGGGAGGAGCTAGG | NM_001285986 | 144 | S1 |
| OCT4_R | CTTCCCTCCAACCAGTTGCCCCAAAC |  |  |  |
| PLAU_F | CCATCTACAGGAGGCACCG | NM_001145031.1 | 140 | S14 |
| PLAU_R | GCGACCCAGGTAGACGATG |  |  |  |
| LAMC2_F | GATGGCATTCACTGCGAGAAG | NM_005562.2 | 105 | S15 |
| LAMC2_R | TCGAGCACTAAGAGAACCTTTGG |  |  |  |
| PDPN_F | GGAAGGTGTCAGCTCTGCTC | NM_006474.4 | 114 | S16 |
| PDPN_R | CGCCTTCCAAACCTGTAGTC |  |  |  |
| CCND1_F | TTCGGGATGATTGGAATAGC | NM_053056.2 | 150 | S17 |
| CCND1_R | TGTGAGCTGGCTTCATTGAG |  |  |  |
| VEGF_F | TGCAGATTATGCGGATCAAACC | M32977.1 | 81 | S18 |
| VEGF_R | TGCATTCACATTTGTTGTGCTGTAG |  |  |  |
| TNC_F | AGCTCAACCATCACTGCCAAG | NM_002160.3 | 137 | S19 |
| TNC_R | TAGACCAGCAGGTAACCGGTG |  |  |  |
| CTNNB1_F | GTGCTATCTGTCTGCTCTAGTA | NM_001904.3 | 154 | S20 |
| CTNNB1_R | CTTCCTGTTTAGTTGCAGCATC |  |  |  |
| UPAR_F | CCCAATCCTGGAGCTTGAAA | X51675.1 | 166 | S21 |
| UPAR_R | TTGGTTTTTCGGTTCGTGAGT |  |  |  |
| MYO1B_F | GGTCTGGTGTGGAGGTCCTA | NM_001130158.1 | 127 | S22 |
| MYO1B_R | CGTTGCTTCCTCAGGTCTTC |  |  |  |

**Table S8:** Summary of the primary antibodies used along with their antigen-retrieval conditions

| Primary Antibody | Clone | Manufacturer | Antigen retrieval | | Working dilution |
| --- | --- | --- | --- | --- | --- |
| MMP9 | EP127 | BioGenex Laboratories, CA, U.S.A. | Tris-EDTA buffer (pH-8) in pressure cooker for 10 minutes | | Pre-diluted |
| LAMC2 | Sc-25341 | Santa Cruz, CA, USA | Tris-EDTA buffer (pH-8) in pressure cooker for 10 minutes | | 1:150 |
| ECAD | EP6 | PathnSitu, CA, U.S.A. | | Tris-EDTA buffer (pH-8) in pressure cooker for 10 minutes | Pre-diluted |

**Table S9:** Univariate survival analysis for disease-free and overall survival in patients treated by surgery

|  | **Disease-free Survival** | | **Overall Survival** | |  | |
| --- | --- | --- | --- | --- | --- | --- |
| **Variables** | | **Hazard ratio (95% C.I.)** | | **P value** | | **Hazard ratio (95% C.I.)** |
| Age | | 1.29 (0.44 - 3.77) | | 0.64 | | 0.8 (0.17 - 3.85) |
| Gender | | 0.03 (0 - 2.9) | | 0.13 | | 0.03 (0 - 16.86) |
| Tobacco chewing | | 1.84 (0.67 - 5.07) | | 0.24 | | 2.39 (0.64 - 8.94) |
| Tobacco smoking | | 1.91 (0.68 - 5.38) | | 0.22 | | **5.7 (1.42 - 22.94)** |
| Alcohol | | **4.06 (1.44 - 11.46)** | | **0.008** | | **8.43 (1.74 - 40.77)** |
| Habits | | **1.98 (1.06 - 3.71)** | | **0.03** | | **3.85 (1.29 - 11.45)** |
| Node status | | **3.12 (1.1 - 8.8)** | | **0.03** | | 3.3 (0.88 - 12.35) |
| Stage | | 0.48 (0.06 - 3.65) | | 0.48 | | 0.04 (0 - 205.83) |
| Grade | | 1.49 (0.65 - 3.44) | | 0.35 | | 2.37 (0.79 - 7.14) |
| PNI | | **3 (1.06 - 8.51)** | | **0.04** | | 2.87 (0.77 - 10.75) |
| Occult node status | | **4.09 (1.42 - 11.76)** | | **0.009** | | **4.68 (1.25 - 17.55)** |
| LAMC2 | | 1.87 (0.66 - 5.27) | | 0.24 | | 1.68 (0.45 - 6.28) |
| ECAD at ITF | | **25.39 (3.3 - 195.13)** | | **0.002** | | **10.81 (1.33 - 87.99)** |
| MMP9 | | 4.06 (0.84 - 19.59) | | 0.08 | | 1.41 (0.23 - 8.44) |

**Supplementary References:**

S1. Huang, H.-P. *et al.* Epithelial cell adhesion molecule (EpCAM) complex proteins promote transcription factor-mediated pluripotency reprogramming. *J. Biol. Chem.* **286**, 33520–32 (2011).

S2. Yu, T. *et al.* CXCR4 Promotes Oral Squamous Cell Carcinoma Migration and Invasion through Inducing Expression of MMP-9 and MMP-13 via the ERK Signaling Pathway. *Mol. Cancer Res.* **9**, 161–172 (2011).

S3. Huang, X. *et al.* Analysis of the expression pattern of the BCL11B gene and its relatives in patients with T-cell acute lymphoblastic leukemia. *J. Hematol. Oncol.* **3**, 44 (2010).

S4. Carr, J. R. *et al.* FoxM1 Regulates Mammary Luminal Cell Fate. *Cell Rep.* **1**, 715–729 (2012).

S5. American Association for Cancer Research., International Cancer Research Foundation. & William H. Donner Foundation. *Cancer research : the official organ of the American Association for Cancer Research, Inc.* *Cancer Research* (Waverly Press, 2018).

S6. Barber, A. G. *et al.* Characterization of Desmoglein Expression in the Normal Prostatic Gland. Desmoglein 2 Is an Independent Prognostic Factor for Aggressive Prostate Cancer. *PLoS One* **9**, e98786 (2014).

S7. Abtin, A. *et al.* Flagellin is the principal inducer of the antimicrobial peptide S100A7c (psoriasin) in human epidermal keratinocytes exposed to *Escherichia coli*. *FASEB J.* **22**, 2168–2176 (2008).

S8. Liu, Z. *et al.* RUNX3 regulates vimentin expression *via* miR-30a during epithelial-mesenchymal transition in gastric cancer cells. *J. Cell. Mol. Med.* **18**, 610–623 (2014).

S9. Haynes, K. A. & Silver, P. A. Synthetic reversal of epigenetic silencing. *J. Biol. Chem.* **286**, 27176–82 (2011).

S10. Tanaka, N. *et al.* Expression of carbonic anhydrase 9, a potential intrinsic marker of hypoxia, is associated with poor prognosis in oesophageal squamous cell carcinoma. *Br. J. Cancer* **99**, 1468–1475 (2008).

S11. van Uden, P., Kenneth, N. S. & Rocha, S. Regulation of hypoxia-inducible factor-1alpha by NF-kappaB. *Biochem. J.* **412**, 477–84 (2008).

S12. Li, S.-J., Yang, X.-N. & Qian, H.-Y. Antitumor effects of WNT2B silencing in GLUT1 overexpressing cisplatin resistant head and neck squamous cell carcinoma. *Am. J. Cancer Res.* **5**, 300–8 (2015).

S13. Wang, T. *et al.* Twist2 contributes to cisplatin-resistance of ovarian cancer through the AKT/GSK-3β signaling pathway. *Oncol. Lett.* **7**, 1102–1108 (2014).

S14. Lv, L. *et al.* The DNA methylation-regulated miR-193a-3p dictates the multi-chemoresistance of bladder cancer via repression of SRSF2/PLAU/HIC2 expression. *Cell Death Dis.* **5**, e1402 (2014).

S15. Kim, B. G. *et al.* Laminin-332-Rich Tumor Microenvironment for Tumor Invasion in the Interface Zone of Breast Cancer. *Am. J. Pathol.* **178**, 373–381 (2011).

S16. Kato, Y. *et al.* Aggrus: a diagnostic marker that distinguishes seminoma from embryonal carcinoma in testicular germ cell tumors. *Oncogene* **23**, 8552–8556 (2004).

S17. Jiao, J. *et al.* Cyclin D1 affects epithelial-mesenchymal transition in epithelial ovarian cancer stem cell-like cells. *Onco. Targets. Ther.* **6**, 667–77 (2013).

S18. De Francesco, E. M. *et al.* GPER mediates activation of HIF1α/VEGF signaling by estrogens. *Cancer Res.* **74**, 4053–64 (2014).

S19. Gratchev, A., Kzhyshkowska, J., Utikal, J. & Goerdt, S. Interleukin-4 and dexamethasone counterregulate extracellular matrix remodelling and phagocytosis in type-2 macrophages. *Scand. J. Immunol.* **61**, 10–17 (2005).

S20. Stein, U. *et al.* Intervening in β-catenin signaling by sulindac inhibits S100A4-dependent colon cancer metastasis. *Neoplasia* **13**, 131–44 (2011).

S21. Zhou, H. *et al.* RNAi targeting urokinase-type plasminogen activator receptor inhibits metastasis and progression of oral squamous cell carcinoma *in vivo*. *Int. J. Cancer* **125**, 453–462 (2009).

S22. Chapman, B. V. *et al.* MicroRNA-363 targets myosin 1B to reduce cellular migration in head and neck cancer. *BMC Cancer* **15**, 861 (2015).
